# ARTHROSCOPIC LATARJET WITH CORTICAL BUTTONS: CLINICAL AND RADIOLOGICAL OUTCOMES

**DOI:** 10.1590/1413-785220253303e293793

**Published:** 2025-12-01

**Authors:** Alexandre Tadeu do Nascimento, Caio Santos Checchia, Jorge Henrique Assunção, Mauro Emilio Conforto Gracitelli, Fernando Brandão de Andrade e Silva, Robson Massi Bastos, Arnaldo Amado Ferreira, Eduardo Angeli Malavolta

**Affiliations:** 1 Hospital Orthoservice Sao Jose dos Campos, Grupo de Ombro e Cotovelo, Rede D'or, Sao Paulo, SP, Brazil.; 2 Instituto Trata, Grupo de Ombro e Cotovelo, Sao Paulo, SP, Brazil.; 3 Universidade de Sao Paulo, Faculdade de Medicina, Hospital das Clinicas (HCFMUSP), Sao Paulo, SP, Brazil.; 4 DASA/Hospital 9 de Julho, Sao Paulo, SP, Brazil.; 5 Hospital Sirio-Libanes, Sao Paulo, Brazil.; 6 Hospital do Coracao (Hcor), Sao Paulo, Brazil.

**Keywords:** Shoulder Dislocation, Joint Instability, Orthopedic procedures, Luxação do Ombro, Instabilidade Articular, Procedimentos Ortopédicos

## Abstract

**Objective:**

To evaluate clinical and radiological outcomes of the arthroscopic Latarjet procedure with cortical buttons for traumatic anterior shoulder instability.

**Methods:**

Retrospective case series of medical reports and imaging studies of patients operated between April 2016 and September 2019 at a single hospital. Primary outcome was the Rowe score 24 months after surgery (MCID of 9.7 points). Secondary outcomes were the VAS, Rowe scores at other follow-up points, recurrence of instability, complications and tomographic evaluation of arthritis and graft healing, resorption and positioning.

**Results:**

46 shoulders were evaluated. At 24 months, mean Rowe score increased from 34.4 ± 11.4 to 90.1 ± 12.2 (p<0.001) and VAS from 6.1 ± 2.0 to 1.2 ± 1.9 (p<0.001). Every patient achieved MCID. Over 90% of grafts were well positioned and approximately 85% of them healed. There was no redislocation and only one (2.2%) subluxation. There were complications in six patients (13%), and no reoperation was needed.

**Conclusion:**

At short-term, arthroscopic Latarjet procedure with cortical buttons provides good clinical outcomes, significant pain reduction and low recurrence rate. Graft healing and positioning were adequate. Complications were minor, with no reoperation needed. *Level of Evidence IV; Case Series.*

## RESUMO

**Objetivos:**

Avaliar desfechos clínicos e radiológicos do procedimento de Latarjet artroscópico com botões corticais para instabilidade anterior traumática do ombro.

**Métodos:**

Série de casos retrospectiva de pacientes operados entre abril de 2016 e setembro de 2019 em um único hospital. O desfecho primário foi o escore de Rowe 24 meses após a cirurgia (MCID de 9,7 pontos). Os desfechos secundários foram a EVA, escores de Rowe em outros períodos, recorrência da instabilidade, complicações e avaliação tomográfica de artrose e do enxerto (consolidação, reabsorção e posicionamento).

**Resultados:**

Foram avaliados 46 ombros. O desfecho primário aumentou de 34,4 ± 11,4 para 90,1 ± 12,2 (p<0,001) e a EVA, de 6,1 ± 2,0 para 1,2 ± 1,9 (p<0,001). Todos atingiram o MCID. Mais de 90% dos enxertos estavam bem posicionados e aproximadamente 85% consolidaram. Não houve re-luxação e apenas um caso (2,2%) de subluxação. Ocorreram seis complicações (13%), sem necessidade de reoperação.

**Conclusão:**

A curto prazo, o Latarjet artroscópico com botões corticais proporciona bons desfechos clínicos, redução da dor e pouca recidiva. A consolidação e o posicionamento do enxerto foram adequados, com poucas complicações (sem necessidade de reoperação). *Nível de Evidência IV; Série de Casos*.

## INTRODUCTION

The treatment of recurrent traumatic anterior shoulder instability is preferably surgical.^1^ In cases with increased risk for recurrence, Bankart repair alone is not ideal,^2^ and coracoid transposition procedures (like the Latarjet) lead to reliable results,^3-5^ with significant clinical improvement and few failures.^6,7^

The limitations associated with the open Latarjet procedure have driven the development of arthroscopic techniques, initially introduced by Lafosse et al. in 2007.^8^ Some authors^9-11^ have supported this approach, highlighting advantages such as improved accuracy of graft positioning, reduced risk of neurological injuries, better management of associated lesions, and minimized muscle damage. However, comparative research has not demonstrated a clear advantage of one technique over the other in terms of complication rates, functional outcomes, or radiographic results, leaving the choice of method largely dependent on the surgeon's preference.^12-14^

Similarly, since Gendre et al.^15^ described coracoid process fixation with cortical-buttons (CBs) in 2016, some studies have endorsed this method to mitigate complications linked to screws,^16,17^ while maintaining similar biomechanical strength^18^ and clinical outcomes.^17,19^ Nonetheless, conflicting data on CBs indicate a potential increase in recurrent dislocation.^17,20,21^ and lower biomechanical stability.^22^ However, to date few studies have been published on the use of CBs in Latarjet procedures.^11,19,20,23,24^

This study aimed to analyze clinical and radiologic outcomes after arthroscopic Latarjet with cortical-buttons for traumatic anterior shoulder instability. Our hypothesis is that results would be favorable, with few complications.

## METHODS

This is an institutional ethical committee-approved (CAAE 54388821.5.1001.0068), single-center, retrospective case series study of medical records (after formal signed patient consent), evaluating clinical and radiological outcomes of patients undergoing arthroscopic Latarjet procedures with cortical buttons.

### Data collection

Data from medical reports was retrospectively retrieved by the main author in 2023. Rowe scores and VAS had already been performed by a research assistant, unrelated to this study, when patients were followed at clinic: one week before surgery, and at 3, 6, 12 and 24 months after that. Likewise, pre- and postoperative (at 6 months) CT scans had already been performed.

### Patients

Skeletally mature patients with traumatic anterior shoulder instability associated with any of the following criteria were indicated to surgery: Instability Severity Index Score (ISIS) ≥ 4, anterior glenoid bone loss greater than 20%, off-track injury^25^ or recurrence of instability after Bankart repair.

Patients were operated on at a single institution by a single surgeon, with 11 years of experience, between April 2016 and September 2019.

Inclusion criteria in this retrospective study were: patients with pre- and postoperative clinical and imaging evaluations; a minimum follow-up of two years. Non-inclusion criteria were: multi- or bidirectional instability; one single episode of anterior shoulder dislocation; associated rotator cuff tears or fractures (other than the anterior glenoid rim or Hill-Sachs).

### Standard of Care and Surgical Technique

Anesthesia, surgical technique and rehabilitation were performed in the same manner for every patient, as follows.

Intravenous general anesthesia was used after interscalene nerve block. Antibiotic prophylaxis with Cefazolin (2g every 8 hours for 24 hours) was performed.

**Figure 1:**
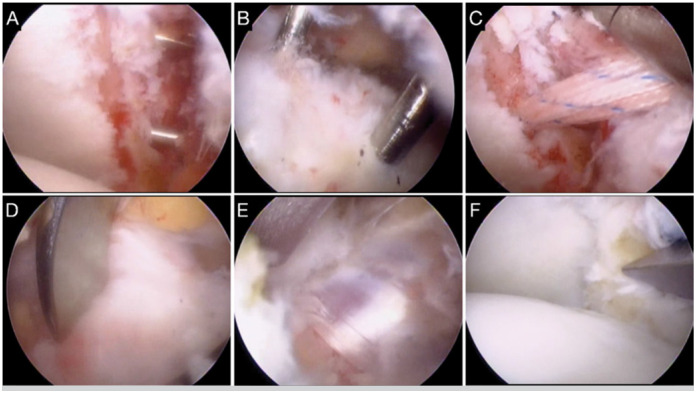
Right shoulder. Intraoperative view from the anterolateral portal. A: Guide wires passed through the glenoid. B: Guide wires passed through the coracoid process. C: Cortical buttons and wires passing through the glenoid tunnels. D: Osteotomy of the coracoid process. E: Conjoint tendon passed through the subscapularis split. F: Final aspect after graft fixation.

Source: Author.

Patients were operated in a "relaxed beach chair" position. Intra-articular inspection was initially performed through the posterior portal. The anterior portal was made above the subscapularis tendon. The anteroinferior labrum, the middle glenohumeral ligament and the anterior capsule were resected to facilitate later graft passage. The anterior glenoid neck was debrided and flattened with shavers. The coracoacromial ligament was released from the coracoid. Viewing from the *J portal* (performed with an *outside-in* technique at tip of the coracoid), the pectoralis minor muscle was detached and the lower surface of the coracoid was flattened. Any adhesion between the axillary nerve and the conjoint tendon was released. The subscapularis was longitudinally split between its superior two-thirds and its inferior one-third. Next, four guide wires were passed (two through the glenoid and two through the coracoid). At the glenoid, guide wires were placed using an anterior cruciate knee ligament reconstruction guide around the shoulder, with its anterior tip placed through the anterolateral portal (positioned at the anteroinferior glenoid neck, 5 mm medial from the articular surface) and its posterior end through the posterior portal. Perforation was performed from posterior to anterior. The same was repeated for the second glenoid guide wire, 2 cm more proximally (Figure 1A). A portal superior to the coracoid base (*H portal*) was created, and two guide wires were passed (approximately 2 cm apart) through the coracoid without any guide (Figure 1B). Using a 4 mm diameter cannulated drill, the first guide-wire (at the inferior glenoid neck) a tunnel was perforated, through which a prolene thread was passed from posterior to anterior, bringing along with it a CB (ToggleLoc^TM^, Biomet - loaded with a number-two high resistant braid). It was passed through the subscapularis split and then temporarily retrieved through the anterolateral portal. The coracoid was tunneled through the most distal guide wire, and the same CB was transported through the coracoid and outside the *H portal*. The same process was repeated through the other two guide wires (Figure 1C). With both buttons placed over the coracoid, osteotomy was performed with a chisel (Figure 1D) and the braids were pulled from the posterior portal. This would bring the coracoid graft, through the subscapularis split, onto the antero-inferior glenoid neck (Figure 1E). Finally, the braids of both CBs were tensioned and sutured over a single posterior CB (Figure 1F). The CBs used had self-locking mechanisms, granting greater graft compression.

Patients wore a sling for 21 days. Passive shoulder range of motion (ROM) started at 14 days, while active ROM, at 21 days. Isometric strengthening started at 30 days and active-resistance exercises, at 45 days. Sports requiring upper limbs and manual labor were allowed after 4 to 6 months, after complete recovery of ROM and strength.

### Methods of Assessment

The primary outcome was the ROWE score at 24 months, for which the MCID value of 9.7 was used.^26^ Other dependent variables were: (1) ROWE score at other periods; (2) VAS for pain; (3) recurrence of dislocation and/or subluxation; (4) graft healing, reabsorption, and vertical and horizontal positioning at CT; (3) complications: infection, neurologic lesion, graft migration and fracture, and glenohumeral arthritis.

Independent variables were: (1) intrinsic to patients: age, sex, dominance, smoking, previous Bankart repair, sports activity and ISIS; (2) related to the injury: glenoid bone loss, Hill Sachs interval, and glenoid tracking pattern.

The main author retrospectively analyzed the CTs, as follows: glenoid bone loss was measured using the *best-fit circle* method;^27^ humeral bone loss by the *Hill-Sachs interval;* ^25^ shoulders were classified as *on-* or *off-track;* ^25^ graft healing, resorption and positioning, as well as glenohumeral arthritis, were also analyzed.

### Statistical Analysis

Continuous variables were assessed for normality using the Kolmogorov-Smirnov test, and homogeneity, the Levene test. Continuous variables are presented as mean, standard deviation, median and interquartile range (IIQ), as continuous data (except "age") had non-parametric distribution. Categorical variables were displayed in absolute values (and %). For functional results comparison (Rowe and VAS scores) over different evaluation periods, the Friedman test was used, and the Wilcoxon test for post-hoc analysis. The SPSS 21.0 was used, with a significance level of 5%.

## RESULTS

During the studied period, 48 patients underwent surgery. Two cases were not included: a rotator cuff tear and a primary dislocation. The series consisted then of 46 shoulders (45 patients).

The mean age at surgery was 32.1 ± 9.3 years. Most patients were male, operated the dominant shoulder, and performed recreational sports regularly. Around 28% had a previous single Bankart repair (Table 1).

**Table 1:** Baseline Characteristics of Patient-Related Variables.

	n	%
Male	39	84.8
Dominant side affected	31	67.4
**Tobacco use**		
Smoker	2	4.3
Former smoker	6	13.0
Epilepsy	1	2.2
Sports activity	37	80.4
Competitive	9	19.6
Collision	14	30.4
Contact	10	21.7
Non-contact	13	28.3
Prior Bankart repair	13	28.3

Average glenoid bone loss was 15.4 ± 10.3%, and Hill-Sachs interval was 15.9 ± 6.1 mm. Nineteen (41.3%) shoulders were *off-track*. Average ISIS score was 5.3 ± 1.6 (Table 2).

**Table 2:** Preoperative Bone Loss and ISIS Score.

	Average	SD	Median	IIQ
Glenoid width (mm)	28.5	2.4	28.5	4.0
Glenoid defect (mm)	4.3	2.9	5.3	7.0
Glenoid % loss	15.4	10.3	19.6	23.0
Hill-Sachs interval	15.9	6.1	16.2	7.0
ISIS score	5.3	1.6	5.0	1.0

**Figure 2:**
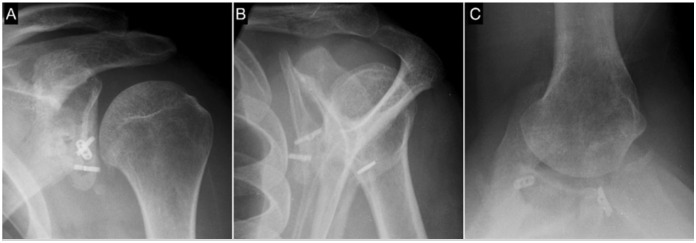
This boxplot shows the distribution of Rowe Scale scores at different evaluation periods. The box represents the interquartile range (IQR), the line inside the box indicates the median, and the whiskers extend to the minimum and maximum non-outlier values. Outliers are shown as individual points.

Source: Author.

**Figure 3:**
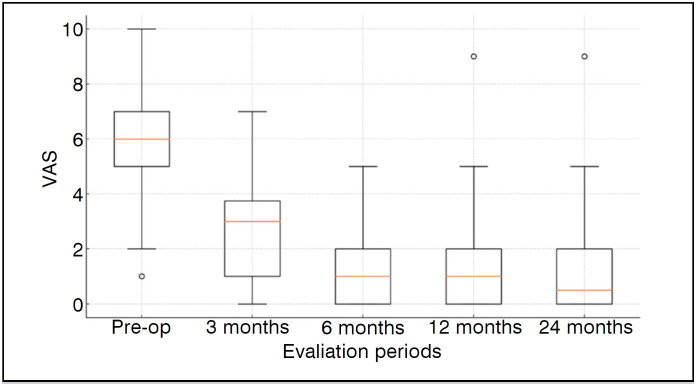
This boxplot presents the distribution of Visual Analog Scale (VAS) scores across different evaluation periods. The box illustrates the interquartile range (IQR), with the median marked by a line inside the box. Whiskers represent the range, and outliers are plotted as individual points.

Outcomes achieved statistically significant improvements. Mean Rowe score increased from 34.4 ± 11.4 to 90.1 ± 12.2 at 24 months (p<0.001). Mean VAS went from 6.1 ± 2.0 to 1.2 ± 1.9 in the same period (p<0.001) (Table 3) (Figures 2 and 3). Every patient achieved MCID.

**Table 3:** Pre and Postoperative Functional Assessment.

	Average	SD	Median	IIQ	P value
**Rowe Score**					
Pre-operative	34.4	11.4	40.0	15.0	<0.001*
3 months	82.2	16.2	87.5	10.0	
6 months	88.2	14.2	90.0	8.0	
12 months	89.6	14.0	92.5	5.0	
24 months	90.1	12.2	92.5	5.0	
**Visual Analogue Score (pain)**					
Pre-operative	6.1	2.0	6.0	2.0	<0.001**
3 months	2.5	1.8	3.0	3.0	
6 months	1.3	1.4	1.0	2.0	
12 months	1.3	1.8	1.0	2	

General comparisons were performed with the Fredman test. Post hoc analyses, with the Wilcoxon test. SD: standard deviation; IIQ: interquartile range;

* post-hoc analysis: difference between all follow-up times, except 12 and 24 months;

** post-hoc analysis: difference between all follow-up times, except 6 and 12 months, 6 and 24 months, and 12 and 24 months.

**Figure 4:**
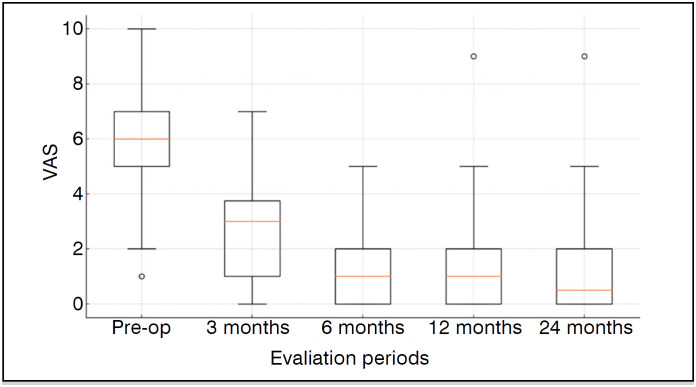
Center shoulder. Radiographs at 24 months postoperative depicting adequate graft healing and no radiographic complication. A: Grashey AP view. B: Scapular profile view. C: Axillary view.

**Figure 5:**
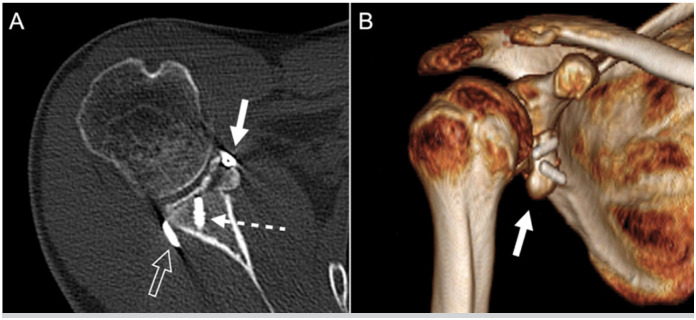
Right shoulder. Computed tomography at 24 months after surgery depicting adequate graft positioning (white arrow). A: Axial view; Hollow arrow points to the large posterior cortical button; Dashed arrow points to a metallic anchor used in the Bankart repair (previous failed surgery). B: Tridimensional reconstruction.

Source: Author.

More than 90% of grafts were well positioned horizontally and vertically, and approximately 85% had healed (Figures 4 and 5). Glenohumeral arthritis, previously observed in two patients (4.3%), was observed postoperatively in four (8.7%) (all Samilson and Prieto stage I) (Table 4).

**Table 4:** Postoperative Tomographic Evaluation.

	n	%
**Glenohumeral arthritis**		
Pre-operative	2	4.3
Post-operative	4	8.7
Graft resorption	4	8.7
**Graft vertical positioning**		
At the equator	4	8.7
Below the equator	42	91.3
**Horizontal positioning**		
Lateralized > 5mm	2	4.3
Well positioned	44	95.7
Healing	39	84.8

There was one subluxation and one positive anterior apprehension test (2.2%), both in the same patient, and no redislocation. We observed six complications (six patients; 13%), including two superficial postoperative infections (4.3%) which were successfully treated with oral antibiotics (Table 5). Complications did not impact clinical outcomes.

**Table 5:** Recurrences and Complications.

	n	%
**Recurrences**		
Dislocation	0	0
Subluxation	1	2.2
**Complications**		
Superficial infection	2	4.3
Graft migration	2	4.3
Axillary neuropraxia	1	2.2
Intraoperative graft fracture	1	2.2

## DISCUSSION

Our results show that traumatic anterior shoulder instability can be successfully treated by the arthroscopic Latarjet procedure with cortical buttons (CBs), which yields good clinical outcomes, significant pain improvement and low recurrence rate.

At 24 months, every patient achieved MCID. Mean Rowe score was 90.1, in accordance with other authors.^9,11,28^ Boileau et al.^9^ in a series of 136 patients with CBs, reported an average of 90 points at 24 months. Girard et al.^11^ with a minimum follow-up of 12 months, achieved 94 points, while Song et al.^28^ average follow-up of three years, 95 points.

We observed significant improvement in pain (VAS improved from 6.1 to 1.2), similar to Song et al. ^28^, (5.3 to 1.2 points). Hardy et al.^20^ assessed pain only at last follow-up, averaging 1.3 point. Others did not assess pain.^9,11^

Our recurrence rate was 2.2%. This is comparable to Boileau et al.^9^ (2.9%), Girard et al.^11^ (0%) and Song et al.^28^ (1.8%). Meanwhile, Hardy et al.^20^ reported a considerably higher rate (8.3%), statistically higher than those undergoing screw fixation (2.5%; p=0.02).

The graft was well positioned in most of our cases, both vertically and horizontally, similar to other authors using CBs.^9,28^ Healing occurred in 84.8%, lower than Boileau et al.^9^ (95%) and Song et al.^28^ (97%). Graft resorption, however, was considerably lower in our series (8.7%) than in Song et al.^28^ (18.5%). We believe variations in healing and resorption rates may result from CB type (with or without self-locking mechanisms), bone bed debridement, and intraobserver variability. However, it does not seem to influence clinical results at 24 months. We also observed an increase in glenohumeral arthropathy frequency from 2.2% to 4.3%, which was not evaluated by other authors who used CBs.

Infection occurred in two cases (4.3%), successfully treated with oral antibiotics alone. Song et al.^28^ reported 1.9% of infections; other authors did not describe it.^9,11,20^ We believe this complication is not due to surgical approach or fixation method, but rather to longer surgical times and/or other population-related factors. Neurological injury occurred in one case (2.2%), lower than Song et al.^28^ (3.7%) and higher than Boileau et al.^9^ (0%) and Girard et al.^11^ (0%). In all studies, neurological changes were only sensitive and resolved spontaneously. Intraoperative graft fracture happened in one case (2.2%) and distal graft migration during follow-up occurred in two cases (4.3%). This is similar to Girard et al.^11^ (4.2%). All other authors^9,20,28^ did not describe graft fracture or migration. None of our patients required reoperation, similar to Hardy et al.^20^ and less than Song et al.^28^ (2%).

Our study has limitations, as follows. Foremost is its retrospective design. The sample size, even if similar to other series, is too small for secondary analyses. The score used (Rowe) is not as sensitive as other scores, such as the WOSI^29^. The postoperative follow-up of two years, even if similar to other studies, is probably insufficient to detect recurrence and progression of arthritis. Isokinetic assessment of patients was not performed, which has been shown to be altered after the Latarjet procedure.^30^ Finally, imaging analysis was conducted solely by the main author, lacking intra- and inter-observer analysis. Despite these limitations, we believe innovative graft fixation techniques, such as cortical buttons, warrant further research due to frequent screw-related complications.^6^ Our study contributes to knowledge in this area. Randomized trials with larger samples are needed to assess the method's advantages and disadvantages.

## CONCLUSION

At short-term, arthroscopic Latarjet procedure with cortical buttons provides good clinical outcomes for the treatment of traumatic anterior shoulder instability. There was significant improvement to Rowe scores (p<0.001), significant pain (VAS) reduction (P<0.001) and a low recurrence rate (n=1; 2.2%). Coracoid graft healing and positioning were adequate. Complications were minor (no clinical consequence) and no reoperation was needed.

## References

[1] Handoll HH, Almaiyah MA, Rangan A (2004). Surgical versus non-surgical treatment for acute anterior shoulder dislocation. Cochrane Database Syst Rev.

[2] Burkhart SS, De Beer JF, Barth JR, Cresswell T, Roberts C, Richards DP (2007). Results of modified Latarjet reconstruction in patients with anteroinferior instability and significant bone loss. Arthroscopy.

[3] Gordins V, Hovelius L, Sandström B, Rahme H, Bergström U (2015). Risk of arthropathy after the Bristow-Latarjet repair: a radiologic and clinical thirty-three to thirty-five years of follow-up of thirty-one shoulders. J Shoulder Elbow Surg.

[4] Longo UG, Loppini M, Rizzello G, Ciuffreda M, Maffulli N, Denaro V (2014). Latarjet, Bristow, and Eden-Hybinette procedures for anterior shoulder dislocation: systematic review and quantitative synthesis of the literature. Arthroscopy.

[5] Amado Ferreira A, Conforto Gracitelli ME, Assunção JH, Brandão de Andrade E, Silva F, Prieto Chang VY, Angeli Malavolta E (2024). Clinical and radiological evaluation of the Bristow-Latarjet procedure in patients with 30 or more years of follow-up. JSES Int.

[6] Gilat R, Haunschild ED, Lavoie-Gagne OZ, Tauro TM, Knapik DM, Fu MC (2021). Outcomes of the Latarjet Procedure Versus Free Bone Block Procedures for Anterior Shoulder Instability: A Systematic Review and Meta-analysis. Am J Sports Med.

[7] Malavolta EA, Souza JAB, Assunção JH, Gracitelli MEC, Silva FBAE, Ferreira AA (2023). TREATMENT OF RECURRENT ANTERIOR SHOULDER DISLOCATION USING THE LATARJET TECHNIQUE. Acta Ortop Bras.

[8] Lafosse L, Lejeune E, Bouchard A, Kakuda C, Gobezie R, Kochhar T (2007). The arthroscopic Latarjet procedure for the treatment of anterior shoulder instability. Arthroscopy.

[9] Boileau P, Saliken D, Gendre P, Seeto BL, d'Ollonne T, Gonzalez JF (2019). Arthroscopic Latarjet: Suture-Button Fixation Is a Safe and Reliable Alternative to Screw Fixation. Arthroscopy.

[10] Valsamis EM, Kany J, Bonnevialle N, Castricini R, Lädermann A, Cunningham G (2020). The arthroscopic Latarjet: a multisurgeon learning curve analysis. J Shoulder Elbow Surg.

[11] Girard M, Dalmas Y, Martinel V, Mansat P, Bonnevialle N (2022). Arthroscopic Latarjet With Cortical Buttons Versus Open Latarjet With Screws: A Short-Term Comparative Study. Am J Sports Med.

[12] Hurley ET, Lim Fat D, Farrington SK, Mullett H (2019). Open Versus Arthroscopic Latarjet Procedure for Anterior Shoulder Instability: A Systematic Review and Meta-analysis. Am J Sports Med.

[13] Cho CH, Na SS, Choi BC, Kim DH (2023). Complications Related to Latarjet Shoulder Stabilization: A Systematic Review. Am J Sports Med.

[14] Cerciello S, Corona K, Morris BJ, Santagada DA, Maccauro G (2019). Early Outcomes and Perioperative Complications of the Arthroscopic Latarjet Procedure: Systematic Review and Meta-analysis. Am J Sports Med.

[15] Gendre P, Thélu CE, d'Ollonne T, Trojani C, Gonzalez JF, Boileau P (2016). Coracoid bone block fixation with cortical buttons: An alternative to screw fixation?. Orthop Traumatol Surg Res.

[16] Xu J, Liu H, Lu W, Deng Z, Zhu W, Peng L (2020). Modified Arthroscopic Latarjet Procedure: Suture-Button Fixation Achieves Excellent Remodeling at 3-Year Follow-up. Am J Sports Med.

[17] Maguire JA, Dhillon J, Sarna N, Keeter C, Scillia AJ, McCulloch PC (2024). Screw Fixation for the Latarjet Procedure May Reduce Risk of Recurrent Instability but Increases Reoperation Rate Compared to Suture-Button Fixation: A Systematic Review. Arthroscopy.

[18] Minuesa-Asensio A, García-Esteo F, Mérida-Velasco JR, Barrio-Asensio C, López-Fernández P, Aramberri-Gutiérrez M (2020). Comparison of Coracoid Graft Position and Fixation in the Open Versus Arthroscopic Latarjet Techniques: A Cadaveric Study. Am J Sports Med.

[19] Metais P, Clavert P, Barth J, Boileau P, Brzoska R, Nourissat G (2016). Preliminary clinical outcomes of Latarjet-Patte coracoid transfer by arthroscopy vs. open surgery: Prospective multicentre study of 390 cases. Orthop Traumatol Surg Res.

[20] Hardy A, Sabatier V, Schoch B, Vigan M, Werthel JD, Study Investigators (2020). Latarjet with cortical button fixation is associated with an increase of the risk of recurrent dislocation compared to screw fixation. Knee Surg Sports Traumatol Arthrosc.

[21] Thamrongskulsiri N, Limskul D, Tanpowpong T, Kuptniratsaikul S, Itthipanichpong T (2023). Clinical Outcomes, Union Rates, and Complications of Screw Versus Button Fixation in the Bristow-Latarjet Procedure for Anterior Shoulder Instability: A Systematic Review and Meta-Analysis. Clin Orthop Surg.

[22] Williams RC, Morris RP, El Beaino M, Maassen NH (2020). Cortical suture button fixation vs. bicortical screw fixation in the Latarjet procedure: a biomechanical comparison. J Shoulder Elbow Surg.

[23] Huish EG, Kelly SR, Cutter BM (2022). Factors affecting biomechanical strength of Latarjet constructs: A systematic review and meta-regression. Shoulder Elbow.

[24] Nascimento AT, Checchia CS, Assunção JH, Gracitelli MEC, Andrade-Silva FB, Bastos RM (2025). Latarjet procedure: open with screws or arthroscopic with cortical buttons? A retrospective cohort comparison of outcomes and complications. J Shoulder Elbow Surg.

[25] Di Giacomo G, Itoi E, Burkhart SS (2014). Evolving concept of bipolar bone loss and the Hill-Sachs lesion: from "engaging/non-engaging" lesion to "on-track/off-track" lesion. Arthroscopy.

[26] Park I, Lee JH, Hyun HS, Lee TK, Shin SJ (2018). Minimal clinically important differences in Rowe and Western Ontario Shoulder Instability Index scores after arthroscopic repair of anterior shoulder instability. J Shoulder Elbow Surg.

[27] Itoi E, Lee SB, Amrami KK, Wenger DE, An KN (2003). Quantitative assessment of classic anteroinferior bony Bankart lesions by radiography and computed tomography. Am J Sports Med.

[28] Song Q, Zhang S, Cheng X, Xiao J, Lin L, Liu Q (2022). Clinical and Radiographic Outcomes After Arthroscopic Inlay Bristow Surgery With Screw Versus Suture Button Fixation: A Comparative Study of 117 Patients With 3.3-Year Follow-up. Orthop J Sports Med.

[29] Kirkley A, Griffin S, McLintock H, Ng L (1998). The development and evaluation of a disease-specific quality of life measurement tool for shoulder instability. The Western Ontario Shoulder Instability Index (WOSI). Am J Sports Med.

[30] Malavolta EA, Cruz DG, Gracitelli MEC, Assunção JH, Andrade-Silva FB, Andrusaitis FR (2020). Isokinetic evaluation of the shoulder and elbow after Latarjet procedure. Orthop Traumatol Surg Res.

